# Surgical telepresence: the usability of a robotic communication platform

**DOI:** 10.1186/1749-7922-7-S1-S11

**Published:** 2012-08-22

**Authors:** Antonio Marttos, Fernanda M Kuchkarian, Emmanouil Palaios, Daniel Rojas, Phillipe Abreu-Reis, Carl Schulman

**Affiliations:** 1 University of Miami Miller School of Medicine Surgery Department (D40), PO Box 016960 Miami, FL 33101, USA; 2 Universidade Federal do Parana, Rua XV de Novembro, 1299, CEP 80.060-000 .Curitiba, PR Brasil

## Abstract

**Introduction:**

The benefits of telepresence in trauma and acute surgical care exist, yet its use in a live, operating room (OR) setting with real surgical cases remains limited.

**Methods:**

We tested the use of a robotic telepresence system in the OR of a busy, level 1 trauma center. After each case, both the local and remote physicians completed questionnaires regarding the use of the system using a five point Likert scale. For trauma cases, physicians were asked to grade injury severity according to the American Association for the Surgery of Trauma (AAST) Scaling System.

**Results:**

We collected prospective, observational data on 50 emergent and elective cases. 64% of cases were emergency surgery on trauma patients, almost evenly distributed between penetrating (49%) and blunt injuries (51%). 40% of non-trauma cases were hernia-related. A varied distribution of injuries was observed to the abdomen, chest, extremities, small bowel, kidneys, spleen, and colon. Physicians gave the system high ratings for its audio and visual capabilities, but identified internet connectivity and crowding in the operating room as potential challenges. The loccal clinician classified injuries according to the AAST injury grading system in 63% (n=22) of trauma cases, compared to 54% (n=19) of cases by the remote physicians. The remote physician cited obstruction of view as the main reason for the discrepancy. 94% of remote physicians and 74% of local physicians felt comfortable communicating via the telepresence system. For 90% of cases, both the remote and local physicians strongly agreed that a telepresence system for consultations in the OR is more effective than a telephone conversation.

**Conclusions:**

A telepresence system was tested on a variety of surgical cases and demonstrated that it can be an appropriate solution for use in the operating room. Future research should determine its impact on processes of care and surgical outcomes.

## Introduction

Telemedicine extends the reach of trauma and surgical care specialists in real-time and regardless of distance, yet its widespread adoption remains elusive**.** Currently healthcare and market forces are driving the demand for innovative solutions to address the discrepancies in access to quality care and patient outcomes. Trauma remains a leading cause of death worldwide; nevertheless the number of trauma specialists continues to decline. Researchers estimate that there will be a 7% deficit in general surgeons by 2020, and close to 20% by 2050 [1]. It is estimated that two billion people have no access to even basic surgical care [2]. Moreover many parts of the world lack access to trauma care, such as in rural areas and austere environments [3]. Simultaneously, rapid evolution of new surgical techniques and procedures has created the necessity for physicians to maintain their knowledge base current and quickly access training and continuing education opportunities. However, travel and logistics can become an impediment, and other cost-effective solutions may be a better option.

Due to technological advances and declines in cost, telemedicine for trauma and surgical care is becoming increasingly a viable option to address these current challenges and demands.

Telemedicine is generally thought of as the utilization of telecommunications and information technologies in providing health care at a distance. Not a novel concept, examples can be dated back to the 1960s when the first surgical case was broadcasted overseas through videoconferencing for educational purposes [4]. Today, telemedicine can facilitate the mentoring of less experienced surgeons remotely, known as telementoring, as well as transfer information between clinicians for consultation purposes. Teleconsultation can be particularly useful for physicians needing to obtain a second opinion from remote medical specialists. Access to remote specialists may also help in patient transfer decision-making, helping distant hospitals treat patients locally when possible by bringing the specialist to the patient. This potentially can improve patient outcomes and safety; while reducing the need for costly, unnecessary transfers.

Although promising, before implementing new technologies it is crucial that the chosen system be appropriately evaluated. For the past two years, the University of Miami Miller School of Medicine has been testing different mobile telemedicine solutions in the operating room of a large, urban level 1 trauma center. The Ryder Trauma Center at Jackson Memorial Hospital is the only level 1 trauma center serving all residents of Miami-Dade County. The primary objective of this study is to ascertain the usability and feasibility of a remote presence robot for use in the operating room during real surgical cases. The goal is to determine the strengths and weaknesses to its implementation for future telementoring and consultation purposes.

## Materials and methods

### Study design

**Figure 1:**
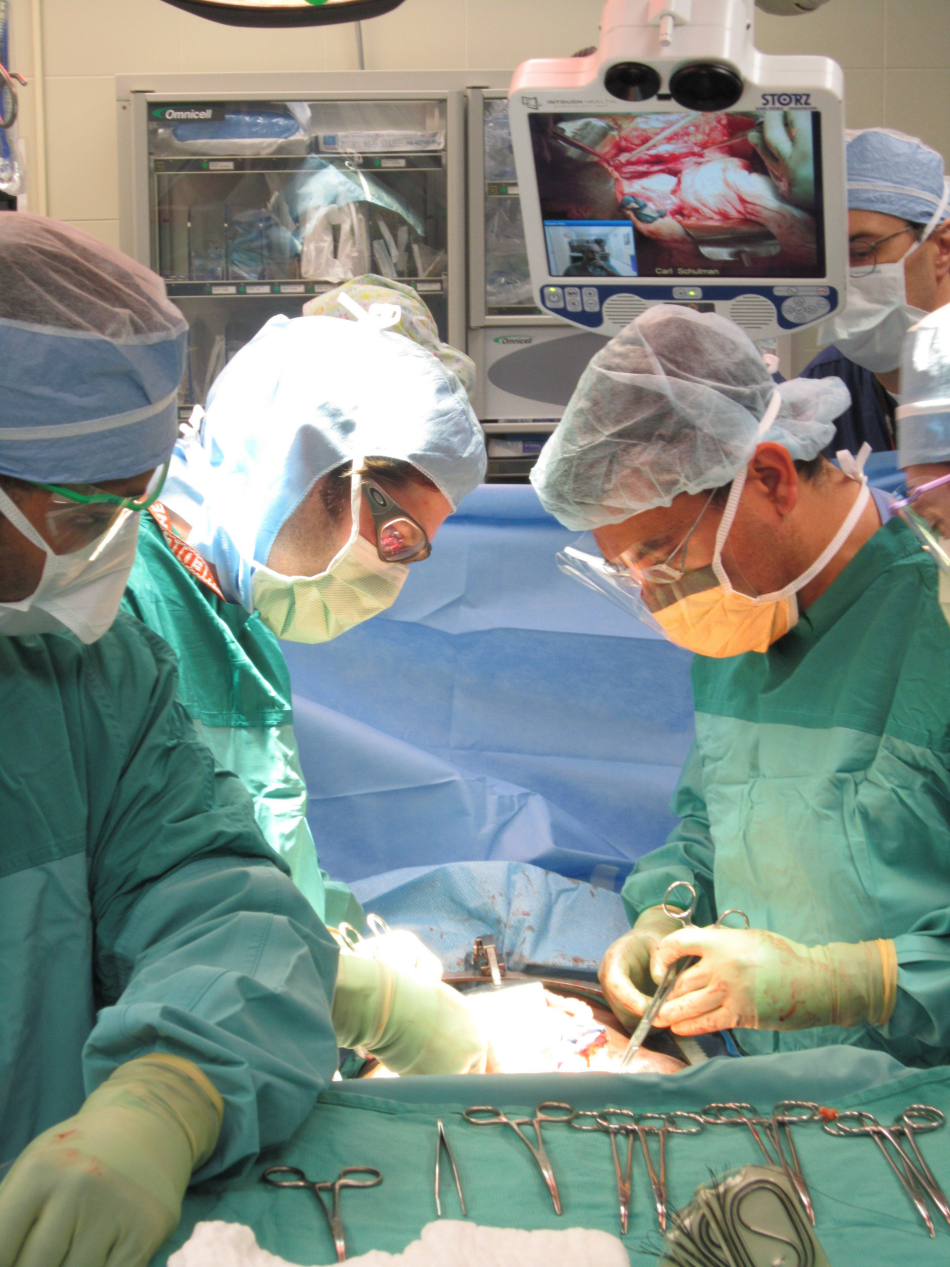
The VisitOR1™ adjustable height gives the remote specialist a view of the surgical field, allowing for consultation and interactive mentoring in real-time with the local on-site surgeons.

We collected prospective, observational data regarding the usability of a telepresence robot in the operating room (Figure 1). Data was collected on 50 surgical cases over a 4 month period from December 2010 to March 2011. We included both trauma and non-trauma surgical cases. Once notified of a case, the robot was wheeled into the operating room by a member of the research team. From a remote location in the hospital - an office on the second floor- the remote physician connected to the robot to see the activities in the operating room and communicate with local clinicians. From the remote location the physician can control the camera (pan, tilt and zoom) to get the best angle of the procedure. At the end of the surgical procedure, both the remote and local physicians are surveyed on their perceptions of using the telepresence robot.

### Participants

Participants included trauma center attending physicians and fellows. Prior to the study, physicians were notified about the telemedicine robot and the study via a study memo. Physicians who were interested in participating received a briefing from the research team and gave consent verbally to participate. Survey data was collected anonymously. No patient data was collected. Physicians received a short training on how to maneuver the robot prior and a member of the research team was present at all times to ensure that the research did not interfere with standard clinical activities.

### Technology

**Figure 2:**
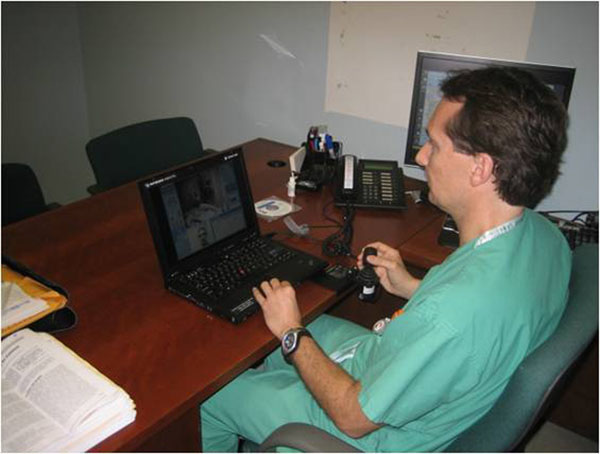
The VisitOR1™ can be remotely operated with through a portable, laptop ControlStation that is linked via the Internet over a secure broadband connection.

The Karl Storz-InTouch VISITOR1™ system is an intraoperative, spring arm mounted communications platform comprised of a ControlStation and Robot. The ControlStation and Robot are linked via the Internet over a secure broadband connection. Through the ControlStation, either installed on a laptop or desktop, a remote physician can gain access to the OR from home or office (Figure 2). The system communicates bi-directionally using TCP and/or UDP, and requires outbound HTTP access to connect to the In Touch Health servers. The VISITOR1 System incorporates encryption methodology utilizing a combination of RSA public/private key and 128-bit AES symmetric encryption.

### Survey

The survey consisted of mainly usability and technical questions, as well as some descriptive questions about the surgical procedure. Responses were rated using a 5-point Likert scale. Survey questions were pretested among a similar study population in a previous pilot study. Examples of technical questions include audio/visual capabilities as well as ease of operation of the robot. An independent observer was present in the operating room to ensure the robot did not interfere with the OR activities. In addition to the usability and technical information of the equipment, we also added some questions regarding the ability of the remote physician to grade the injuries observed. Clinicians were given a copy of the American Association for the Surgery of Trauma (AAST) Scaling System for Organ Specific Injuries [5] Tables as a guide. Grading scales exist for the following organ systems: Cervical Vascular Injury, Chest Wall Injury, Heart Injury, Lung Injury, Thoracic Vascular Injury, Diaphragm Injury, Spleen Injury, Liver Injury, Extrahepatic Billiary Tree Injury, Pancreas Injury, Esophagus Injury, Stomach Injury, Duodenum Injury, Small Bowel Injury, Colon Injury, Rectum Injury, Abdominal Vascular Injury, Adrenal Organ Injury, Kidney Injury, Ureteral Injury, Bladder Injury, Urethra Injury, Uterus (non-pregnant) Injury, Uterus (pregnant) Injury, Fallopian Tube Injury, Ovary Injury, Vagina Injury, Vulva Injury, Testis Injury, Scrotum Injury, Penis Injury, Peripheral Vascular Organ Injury.

During the procedure, the remote physician asked the on-site surgeon to expose the injury and was able to ask questions in order to determine the grade of injury for each damaged system. The grade determined by the remote physician was not communicated to the on-site physician, who was then asked to grade all the injuries at the end of the operative procedure. The two grades were compared to determine the accuracy of the remote physician in grading traumatic injuries through the telepresence robot. Descriptive statistics was used to analyze all survey results.

### Institutional Review Board

The study was reviewed and approved by the University of Miami Institutional Review Board, the Jackson Memorial Hospital Clinical Research Review Committee and the Department of Defense Human Research Protection Office.

## Results

Data was collected on 50 surgical cases, both emergency (80%) and elective cases (20%). Patients were classified as trauma (70%) and non-trauma patients (30%). The majority of cases (64%) were emergency surgery on trauma patients, almost evenly distributed between penetrating (49%) and blunt trauma (51%). 40% of non-trauma cases were hernia-related Participants included 13 attending physicians and 9 fellows. There was a varied distribution of injuries and operative anatomical structures (Table 1)

**Table 1:** Injury location distribution

	# of cases		# of cases
**Trauma Patients**		**Non-Trauma Patients**	

Head	1		
Neck		Abdomen	
Larynx	1	Wall	2
		Inguinal Hernia	5
Chest		Ventral Hernia	2
Wall	4	Small bowel	3
Rib	1	Spleen	1
Vena Cava	1		
Subclavian Artery/Vein	2	Inguinal Lymph Node	1
Abdomen		Unspecified	1
Wall	3		
Stomach	1		
Spleen	4		
Bladder	1		
Kidney	1		
Small Bowel	4		
Colon	5		
Unspecified	2		
Extremities	3		
Miscellaneous			
Skin graft	1		

**Figure 3:**
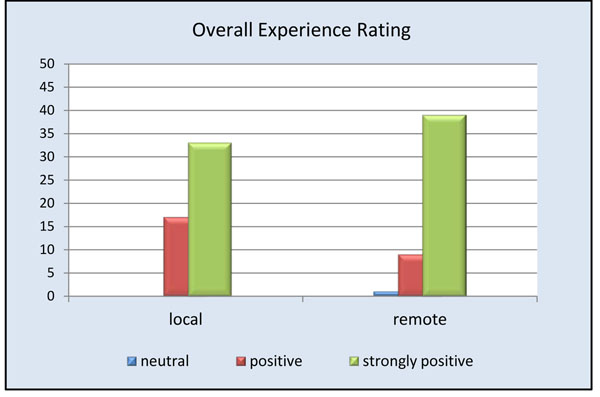
Overall experience using telepresence robot.

**Figure 4:**
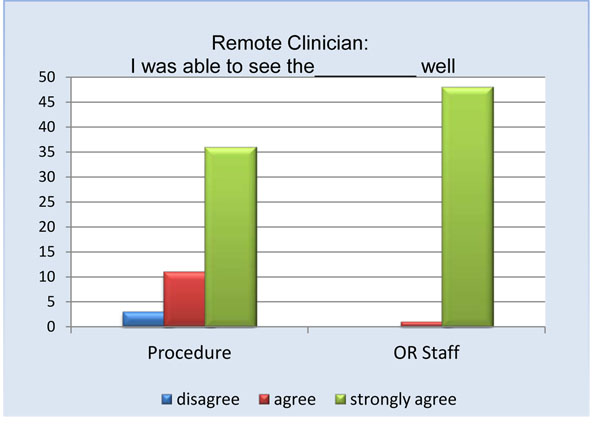
Remote clinician visual ability rating.

**Figure 5:**
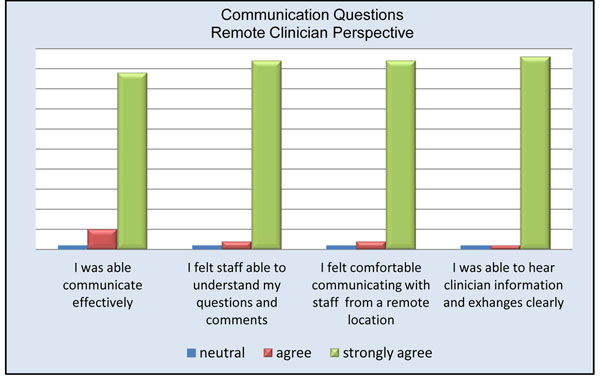
Communication questions remote clinician perspective.

**Figure 6:**
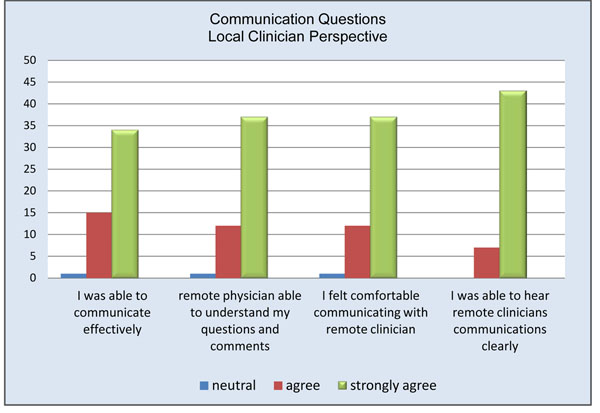
Communication questions local clinician perspective.

**Figure 7:**
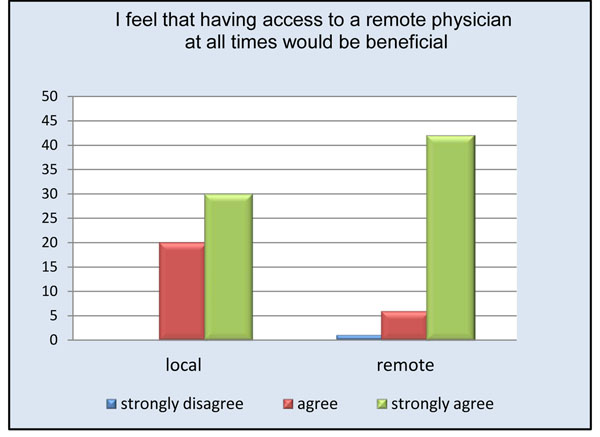
Access to remote physician at all times.

**Figure 8:**
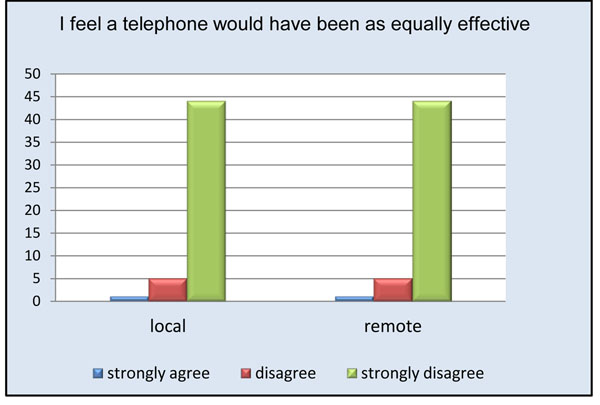
Comparison of telepresence versus telephone.

Remote physicians reported a high level of satisfaction with the use of the telepresence robot (Figure 3). Almost all remote participants (94%) agreed or strongly agreed being able to see the procedure well (Figure 4). The only times the remote clinician noted having difficulties visualizing the procedure occurred when the operating table was surrounded by a team of clinicians. Internet connectivity was an issue in 24% of the cases, ranging from minimal interruption to slow connection speeds. Crowding in the operating room obstructed the view for the remote physician in less than 20% of the cases; however, due to the slim design of the robot it could be moved to either the foot or head of the bed without interference. 94% of remote physicians and 74% of local physicians felt comfortable communicating via the telepresence system (Figures 5 and 6). To measure the value of the telepresence robot, we compared its use to that of the telephone. The most significant finding from the study is that all the local clinicians agreed that having access to a remote expert would be beneficial, and that to do so it would be more effective through telemedicine rather than just the telephone (Figures 7 and 8).

When appropriate, the local clinician used the AAST injury grading system to classify injuries in 63% (n=22) of trauma cases, compared to 54% (n=19) of cases by the remote physicians. In one case, the remote physician reported not being able to differentiate structures such as nerves, arteries or veins due to the amount of blood in the field. In two cases, the remote physician could not grade the injuries due to the overcrowding in the operating room. There was only one case that the remote physician graded one of the injuries, but missed a level III small bowel injury, but the reason was not recorded.

## Discussion

In this observational study, descriptive data was obtained on the use of a robotic telepresence system and its usability inside the operating rooms of a level 1 trauma center. We collected data on 50 surgical cases with the robotic telemedicine system. The majority of the cases were trauma surgical cases, with a few elective general surgery cases. Participants as well as OR staff found the system to be compact and easy to maneuver, which made it more readily acceptable by the operating room staff. The majority of the responses regarding the audio and visual capabilities of the system were highly positive. The only times the remote clinician noted having difficulties visualizing the procedure occurred when the patient was surrounded by a team of clinicians. However, due to the slim design, the cart could be moved to either the foot or head of the bed without interference. Both the local and remote clinicians positively rated the communication abilities and level of comfort using the system. Moreover, the use of a telemedicine system was seen as more beneficial than the traditional phone for consultation purposes. The ability to have the remote expert connect using audio/visual capabilities enhances the experience. We also found that the robot used in this study has sufficient video qualities to allow remote clinicians to see the wounds and organs clearly enough to identify the injury severity.

This study has important limitations. First, a convenience sample was used for the surgical cases. This was done due to several factors, but mainly because the main objective of this study was only to understand the system’s functions, strengths and weaknesses. The main purpose of testing a novel technology is to understand the system’s capabilities as well as how its acceptance can affect the integration of new technology. However, we were able to engage a good number of attendings and fellows to participate to reduce the number of repeat times for any one participant. We were able to capture a variety of injuries and anatomical locations. The results of our study may not be applicable to other hospitals or trauma centers. The results from this study will, however, help guide future efforts. Future directions are to determine if the use of a telepresence system for mentoring and consultation purposes impacts the process and outcomes of care.

## Conclusion

In conclusion, a robotic telepresence system that is mobile and compact in size was readily accepted by the staff in the operating room and physicians. Physicians were able to use the ControlStation with little training or experience. We were able to test the system’s functionalities on a variety of trauma and surgical cases. The potential applications of this system for military and civilian purposes should be further evaluated.

## Competing interests

The authors declare that they have no competing interests.

## Authors' contributions

AM provided the direction and guidance to the research conception and design. FK was involved with the data management and analysis, and drafted the manuscript. EP and DR assisted with the data collection and entry. PA-R assisted with the data interpretation and draft of manuscript. CS assisted with study concept and design, and data interpretation. All authors read and approved the final manuscript.
